# Another Round of “Clue” to Uncover the Mystery of Complex Traits

**DOI:** 10.3390/genes9020061

**Published:** 2018-01-25

**Authors:** Shefali Setia Verma, Marylyn D. Ritchie

**Affiliations:** 1 The Huck Institute of Life Sciences, The Pennsylvania State University, University Park, PA 16802, USA; shefali.setiaverma@pennmedicine.upenn.edu; 2 Department of Genetics, Perelman School of Medicine, University of Pennsylvania, Philadelphia, PA 19104, USA

**Keywords:** complex traits, meta-dimensional analysis, multi-omics datasets, heritability, game of “Clue”

## Abstract

A plethora of genetic association analyses have identified several genetic risk loci. Technological and statistical advancements have now led to the identification of not only common genetic variants, but also low-frequency variants, structural variants, and environmental factors, as well as multi-omics variations that affect the phenotypic variance of complex traits in a population, thus referred to as complex trait architecture. The concept of heritability, or the proportion of phenotypic variance due to genetic inheritance, has been studied for several decades, but its application is mainly in addressing the narrow sense heritability (or additive genetic component) from Genome-Wide Association Studies (GWAS). In this commentary, we reflect on our perspective on the complexity of understanding heritability for human traits in comparison to model organisms, highlighting another round of clues beyond GWAS and an alternative approach, investigating these clues comprehensively to help in elucidating the genetic architecture of complex traits.

## 1. Complex Diseases and the Concept of Heritability

Elucidating the genetic underpinnings of diseases that occur frequently in the population, such as obesity, type 2 diabetes, hypertension, and cancer, are essential research foci among researchers in the human genetics community. The underlying genetic etiologies behind these common human traits are inherently complex due to the effects of multiple genes on the phenotype in comparison to diseases such as cystic fibrosis which follow a Mendelian pattern of inheritance [1]. Remarkably, genetic association studies have been shown to be successful over the past two decades in identifying genetic variants associated with common, complex traits. For example, the first successful Genome-Wide Association Studies (GWAS) identified moderate effect size variants such as *CFH* for age-related macular degeneration (odds ratio 1.25–20.28) [2,3,4], while rare variant association studies have identified multiple rare variants in *ANGPTL4* associated with Coronary Artery Disease (odds ratio 0.32–0.81) [5]. Discovery of many such associations has also impacted the field of pharmacogenomics where variants from these association studies have resulted in drug development and repositioning strategies [6,7]. 

GWAS are mainly performed using retrospective study designs from sample populations collected from either academic medical centers/healthcare provider organizations (examples include eMERGE Network, MyCode Community Health Initiative, and BioVU among others) [8,9,10] or population-based, epidemiological study designs (NHANES, GIANT, CHARGE, etc.) [11,12,13].

**Figure 1:**
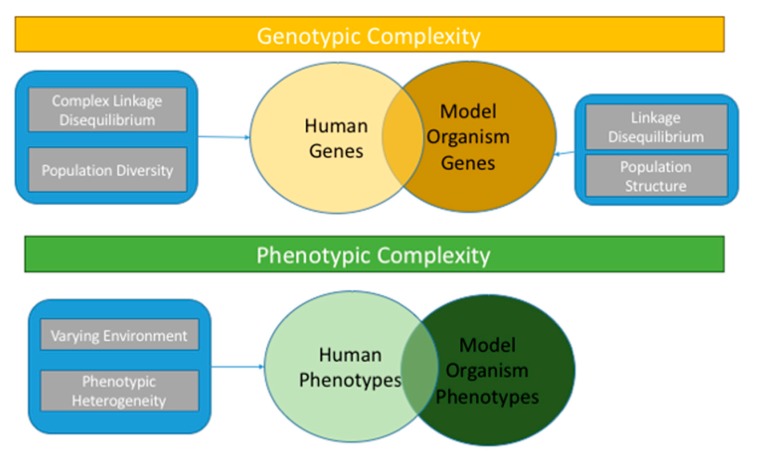
Differences among genotypic and phenotypic complexity in humans and model organisms. The intersection represents orthologous genes (yellow section) and phenotypes (green section).

Despite the success of association analyses among heritable complex diseases, a meager proportion of phenotypic variance has been explained [14,15,16]. Many factors contribute to the unexplained proportion of variance. In most studies, environmental factors and longitudinal effects on the population remain underutilized merely due to unavailability resulting from the strenuous task of collecting these measures [17,18]. Alongside, contributions of additional factors such as structural variations, epistasis, and environmental factors have been proposed as alternative hypotheses for understanding the genetic architecture of complex traits. Comprehensive conventional approaches for validating these hypotheses in model organisms have also shown great success [19,20,21,22,23]. A myriad of approaches are applied for testing the effects of genetic associations in model organisms such as yeast, flies, and mice [24,25,26,27,28]. These model organisms possess human orthologous genes as well as phenotypes that can be directly correlated with human phenotypes [29,30]. Testing of associations in model organisms has helped us in many ways, but the gap between validation of all possible genetic associations and the limited number that have been achieved lies in the potential genetic and phenotypic overlap between humans and model systems. Phenotypic stability among model organisms results in straightforward phenotypic changes due to the low effects of external factors, such as environment, which occasionally makes the concept of missing heritability in humans seem delusional [31]. Validation of associations in model organisms are usually evaluated as quantitative traits but that is not true for all human phenotypes and likewise not all human genes have model organism gene orthologs. Another difference between humans and model organisms lies in the complexity and heterogeneity on both the phenotype and genotype side in humans, whereas model organisms are much simpler. The human genome has much more complex linkage disequilibrium and population diversity than model organisms. In addition, in model organisms, the environment and phenotypes are well controlled in laboratory testing. These differences are depicted in Figure 1.

Heritability of a disease trait refers to the proportion of variance that can be explained by genetic factors. Estimation of heritability is usually done by observing patterns of inheritance among samples either in family based studies or in population based studies. In family based studies, patterns among a pedigree of family members or among monozygotic and dizygotic twins are estimated. In these studies, environmental factors are assumed to be constant. Whereas in population based studies, patterns of inheritance among the population are observed in non-stationery environmental conditions. In this commentary, we highlight studies aimed towards understanding the heritability of complex traits in the realm of sifting through the stack of clues to uncover the mystery of complex trait genetic architecture. We will also emphasize the validation of association analyses from model organisms where available and challenges in interpreting these validated associations.

## 2. Clues to Elucidating the Underlying Genetic Architecture of Complex Traits

Genetic association studies are founded on the bedrock of the concept of heritability. Significant challenges for association studies arise in the realm of answering the following fundamental questions:

1. How much closer do we get to explaining heritability as estimated by family and twin studies by exploiting genetic variations in population based studies?
2. How much of the phenotypic variance is additive (i.e., the combinatorial effect of all variations)? This is referred to as narrow sense heritability.
3. How much of variance cannot be explained merely by adding all variations in a single model?

**Figure 2:**
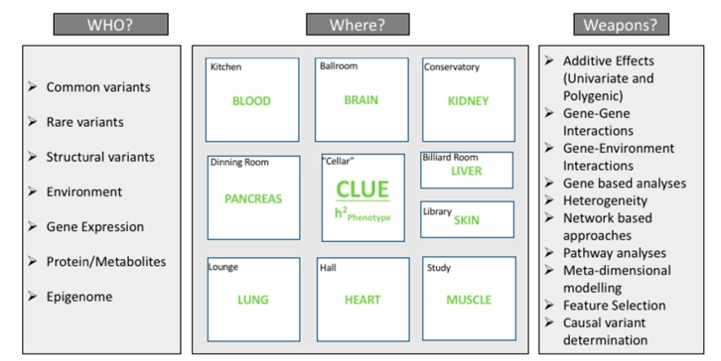
Unriddling undercover heritability. A depiction of the mystery of heritability in the context of the “Game of Clue.” Here, tools and methods to understand heritability are shown as weapons, suspects are genomic elements contributing to heritability, and tissues that are impacted are represented as rooms on the “Clue” game board. The size of rooms does not correspond to importance. This figure is adapted from [43].

Answers to these questions vary from one phenotype to another phenotype. For example, a trait such as height is highly heritable (>90% h^2^_FAM_ (family based estimate of heritability) [32]; offspring resemble their parents) and association studies have shown that the majority of height heritability can be explained by the additive variance components of common variations h^2^_SNP_ (SNP-based estimate of heritability_)_, whereas complex traits such as obesity, type 2 diabetes, and neurological disorders among others are also estimated to be highly heritable (h^2^_FAM_ 47–90%, 20–80%, and 37–81%, respectively) [33,34]. However, association studies using common variants have only explained 27%, 10%, and 21–28% proportion of variance for these traits, respectively [33,35,36]. Considering the predominant explanation for height is through additive genetic variance, GWAS studies have also explained a substantial amount of the genomic heritability for some other traits such as body mass index (BMI), where over 50% of the heritability estimated by family and twin studies has been explained. The same does not stand true for many other binary phenotypes, such as type 2 diabetes, Crohn’s disease, Alzheimer’s disease, etc. There are many clues that may contribute towards the unexplained heritability of these types of complex traits [37,38,39] which include effects due to: (1) rare variations; (2) structural variations; and (3) gene–gene or gene–environment interactions effects among others. Exploring multi-omics data in many studies has also gotten us a bit closer to uncovering the mystery of complex trait architecture [40]. That said, it is still proven difficult to predict which genomic components/elements will be essential for each different complex phenotypic trait. How can we know which elements are responsible for the underlying genetic architecture of each unique trait? To uncover the heritability of common, complex traits, it seems as though most or all these components are playing a different “Game of Clue” in explaining the proportion of phenotypic variance as attributable to genetic/genomic variations. In the following section, we propose our model for understanding this mystery of complex trait architecture by putting the genomic elements contributing to the different effects in the context of “suspects”, types of statistical analysis methods in the context of “weapons”, and which tissue these elements are being tested/evaluated in the context of “rooms” to compare it to the Hasbro board game “*Clue: The Classic Mystery Game*” (Hasbro, Pawtucket, RI, USA). In this classic board game, the goal is to identify who committed a crime, with what weapon the crime was committed, and where the crime took place. A similar analogy has been used in the area of genome sequencing to solve the medical mystery of a drug resistant bacterial spread [41]. This analogy has also been used to describe the mystery of missing heritability [42]; we expand upon this idea in this review. We describe this analogy further in Figure 2. We propose that each common, complex trait should be studied in the same manner that a game of “Clue” would be played. Here, the “crime” is the complex trait risk. In each new round, there is no a-priori hypothesis about who the suspect would be. There is no preconceived notion of the weapon or the room. Similarly, in studies of complex traits, we can expect different genomic and environmental elements to be responsible for disease risk, based on different underlying models, functioning in different tissues. We should not assume that all complex traits will follow a polygenic, additive model simply because height demonstrates this type of model. Other traits may be due to different types of effects, in alternative models, and in different tissues. We discuss all of these components in detail in the following sections.

## 3. Suspects/Who Did It? Considering Different Types of Omics and Environmental Variability as the Suspects in the Crime (Influencing Disease Risk)

### 3.1. Common Variants

Over the past decade, studies to correlate common genetic variations with phenotypic effects have become a standard part of genetic association studies. Among the most popular tools/weapons for investigating common variants are GWAS where single nucleotide polymorphisms (SNPs) obtained via genotyping chips and/or imputed data are tested for association with a phenotype of interest. GWAS have identified over 50 K SNP-trait associations [44,45,46]. GWAS studies are based on patterns of linkage disequilibrium (LD) and assume that tag SNPs in LD with causal SNPs could help in increasing our understanding of complex traits. Analyses of common variants (SNPs) in common diseases follows the common-variant-common-disease (CDCV) hypothesis, which suggests that a combination of common loci could be responsible for common diseases [47,48]. GWAS have been successful in identifying tag SNPs for complex disorders such as Crohn’s disease [49], obesity [50], type 2 diabetes (T2D) [51], multiple sclerosis [52], age-related macular degeneration (AMD) [4] and breast cancer [53] among many others. Many associations identified by GWAS have been validated in model organisms and have also shown pharmacological implications. For example, GWAS have identified variations in the *FTO* gene to be associated with obesity and studies in mouse models also suggest a lean body type when these genetic variations are present [54]. Orthologous phenotypes for metabolic traits such as obesity and T2D are easily captured in model organisms whereas other complex human diseases such as AMD, which is comprised of many factors, are difficult to recapitulate exactly in model organisms [55]. Despite this difficulty, mouse models have suggested an important role of GWAS-identified variants in *CFH* with AMD progression and pharmacological progress has been made regarding the design of a drug targeting *CFH* for AMD patients [6].

Biological processes are complex, and they arise due to the integration of many genes that interact in a pathway to complete a cellular process. Thus, exploiting the interactive effects of common variants is a natural extension in elucidating the complexity of common diseases. Analyses of the non-linear genetic effects of common variants are referred to as epistasis [19,38,56,57]. Non-additive genetic effects can be obtained via statistical methods that may not necessarily correspond to biological epistasis [58]. While additive effects can only explain narrow-sense heritability, exploring dominance and interacting effects could potentially get us closer to the estimated heritability, thus getting one step closer to uncovering the underlying heritability. Significant evidence of epistasis has been demonstrated in model organisms such as *Drosophila melanogaster* and *Saccharomyces cerevisiae* [59,60,61,62]*,* but the role and validation of epistasis for most (but not all) complex human diseases are still hidden due to numerous challenges in discovery and replication of epistatic effects. These challenges are out of the scope of this review but have been discussed previously by many [14,37,63]. We hypothesize that for some complex traits, common variants will act through additive models; where other common variants will act through dominant and epistatic models. We should allow the common variant data to be explored under each of these scenarios to maximize our ability to identify common variants associated with complex traits.

### 3.2. Rare Variants

Genetic variants that are less frequent in the population could have potentially large effects on complex diseases, as illustrated in common-disease-rare-variant (CDRV) hypothesis. The CDRV model is not captured by common variant GWAS designs [48]. Analyzing the single effects of each rare variant is not statistically powerful due to the number of samples sequenced in rare variant association studies (RVAS) [64,65] and the rarity of the genetic variants. Thus, collapsing variants into a gene, pathway, or other variant-set to test the association of the contributions of multiple rare variants in collapsed regions on the phenotype of interest is the most popular method. Many analysis tools have been developed for these analyses such as BioBin [66], Sequence Kernel Association Test (SKAT) [67] , Variant Association Tools [68], and RVTESTS [69]. Several genes containing rare variants have been identified with moderate to high effects on complex traits such as the association of low-frequency variants in *IFIH1* with type 2 diabetes [70] and the association of gain and loss of function (LOF) rare variants in *PCSK9* with low density lipoproteins (LDL) levels [7,71]. The role of *PCSK9* in designing lipid-lowering medications has been implicated in mouse model testing [72,73]. Large-scale studies of multiple rare variants are still in their infancy. However, we expect to see an emergence of rare variant association analyses in the coming years. As these studies emerge, we will develop a better understanding of the role of rare variants in the architecture of common, complex traits.

### 3.3. Structural Variations

Single nucleotide variants (SNVs) as explained in the previous two sections are of great importance in understanding the link between genotype and phenotypes, but large structural variations such as insertions, deletions, translocations, and inversions [74] also play a major role in affecting complex diseases and traits. It has been shown that the human genome consists of multiple recurrent sections of insertions and deletions [75,76]. These variations are rather rare in the population occurring at a frequency of less than 0.05% [77]. Structural variations are commonly referred to as copy number variations (CNV) and these large variations span across one or multiple genes. Given the inherent complexity of genotype and phenotype, it is easy to believe that these large variations which span through many genes could influence a large variety of pathways underlying complex diseases [78]. Since CNV frequency and distribution varies to a large extent in even closely related samples, in population-based studies, a measure of the burden of CNV is sometimes used to associate with diseases and traits. Evidence of association of CNV burden has been shown for neurological and behavioral traits such as Autism Spectrum Disorders [79]. We expect that the evidence for the role of structural variation in complex traits will continue to emerge as the resolution and sensitivity of our molecular technologies continue to improve for structural variant detection.

### 3.4. Environmental Factors

The broad sense heritability (H^2^) of complex diseases can be estimated from both genetic and environmental factors. Heterogeneity in association results could be due to the effects of phenotype interactions with exposure or the interaction of genotypes with exposure. Thus, studying the effect of environment is crucial in understanding biological pathways and mechanisms behind complex traits. Studying the effects of environmental exposures on phenotypes is referred to as environment-wide association study (EWAS) [80,81] and the study of the effects of genetic variants in the context of the environment is performed through gene–environment interaction studies [82,83,84]. Environmental exposures vary across a population-based study to a large extent and thus pose several challenges to gene–environment studies. A comprehensive collection of complex heritable measures is required. Several studies by Patel et al. [80,85] have identified disease-associated exposure factors such as pesticides with type 2 diabetes and effects of heavy metals with serum lipids among others. The effect sizes of the exposures on phenotypes are quite high (odds ratios range from 2–4). Thus, many efforts to measure the exposome are in place to obtain standard global environmental variables on study populations [86]. We hypothesize that gene–environment interactions will explain a great deal of undercover heritability.

### 3.5. Gene Expression

Specific regions of the genome consist of genetic variations that lead to highly heritable variability of gene expression [87]. These regions are known as expression quantitative trait loci (eQTL). The effects of eQTLs on the expression of genes are highly tissue dependent. Thus, testing the effect of eQTLs on diseases or traits is also important because these directly affect the expression of genes. Heritability of gene expression is mediated due to the presence of specific variants (such as eQTLs as explained above). Studies by Moffat et al. [88] and Zhu et al. [89] have identified eQTL signals associated with several complex traits. Even though gene expression as measured by next generation sequencing technologies might not directly explain heritability, it is an important suspect to investigate in elucidating the genetic architecture of complex traits to observe how gene expression influences disease risk (hence the crime in investigation). Various methods to test the effects of gene expression on diseases exist, such as Summary based Mendelian Randomization (SMR), PrediXcan, MetaXcan, and CAVIAR (Causal Variant Identification in Associated Regions) [40,89,90,91]. 

Gene expression data have also been utilized in multi-omics integrative approaches. For example, LaCriox et al. [92] proposed a pyramid approach to test for the effect of SNPs and gene expression on the disease. Kim et al. [93] utilized gene expression data from The Cancer Genome Atlas (TCGA) to predict integrative non-linear effects of SNPs, gene expression, and methylation data on complex cancer phenotypes via an artificial neural network based approach (ATHENA) [94].

Another popular approach to leverage gene expression data to identify how gene expression mediates the effect of genetic variants on diseases and trait is Transcriptome Wide Association Study (TWAS). Gusev et al. [95] showed the efficiency of TWAS in identifying genes associated with anthropometric traits. TWAS methods are still in their early stages. Two types of methods have been suggested in this category: Summary Based Mendelian Randomization (TWAS-SMR) and imputation of the cis-genetic component using SNP information TWAS multi-SNP prediction (TWAS-MP) (e.g., PrediXcan). It is not surprising that gene expression affects disease, but TWAS methods do require more thorough testing to understand the suitability to use these methods to understand various genetic architectures and how these analyses can be validated in model organisms.

### 3.6. Protein/Metabolites

The genetic underpinning of disease phenotypes can be better understood better if we can pinpoint the agitations in normal cellular functions that lead to the disease process. The approaches as mentioned above to identify how DNA variations predispose individuals to disease are important, but they do not get us very close to identifying the underlying mechanisms affecting the phenotype unless the specific causal variants are tested. Amino acid changes can lead to the disruptions of proteins in biological pathways. Testing for the effect of protein variability on human diseases could have potential implications for identifying drug targets, susceptibility to diseases, and also in developing preventive care measures. The effect of proteins can be tested in many different ways: analyzing the effect of protein–protein interactions, the impact of protein complexes, and the effect of metabolites. The field of study to link proteins and their functions to diseases is referred to as proteomics. Biomarkers identified by proteomics are used in developing diagnostic measures for early detection of various types of cancers such as prostate cancer [96,97], as well as monitor progression of ovarian cancer [98,99]. Information on proteins such as metabolites can also be used as filters for pinpointing the underlying etiologies for complex traits. For example, Lee et al. [100] hypothesized that testing the effect of metabolites on metabolic disorders would yield important insights. They utilized a network-based approach to identify connections among multiple metabolic disorders based on circulating metabolites. The protein–protein interactions database is a resource consisting of information on biological interactions among proteins. Sun et al. [101] filtered CNVs to identify epistatic effects among CNVs that mapped to proteins based on the protein–protein interactions database and evaluated the impact of CNV-CNV interactions on the expression of genes.

### 3.7. Epigenome

Germline genetic variations in DNA of an individual do not change significantly during the life course, but the chemical changes in DNA such as methylation and histone modification do change [102,103]. Many factors influence the change in the conformation of DNA structure that could affect underlying cellular mechanisms. Thus, it is also impactful to understand how epigenetic factors influence the disease risk by investigating differences in epigenomes of carriers and non-carriers of diseases. Analysis of the epigenome is mostly studied in the field of cancer genomics to see how these epigenetic factors differ in the cancerous vs. normal tissue [104,105]. Epigenetic changes observed in model organisms can attribute similar biological behaviors in humans because of similar genes in conserved pathways [106]. Thus, the epigenetic analysis is relevant and likely fruitful in model organisms. Model organisms such as yeast and fruit flies, among others, are used to study epigenetic changes such as chromatin structure, DNA methylation, RNA interference (RNAi) pathways, histone modifications [107,108,109,110,111].

## 4. What Is the Weapon of Choice? Which Type of Tools Can Help Elucidate the Significant Risk Factors for Complex Diseases?

In the previous section, we introduced the lineup suspects (or “WHO?”) in the still uncovered mystery of complex trait architecture. These suspects are responsible for influencing disease risk. Another important aspect is to identify which weapons (or “HOW?” the independent variables are modeled) can be used for studying the behaviors of suspects discussed above. Weapons here refer to the tools that can be used by researchers in identifying underlying biological mechanisms. Multiple analysis tools exist in the literature to focus on one or more factors that can contribute towards the susceptibility of complex traits. These weapons or modeling tools are listed in Table 1. Some of these tools have already been described in previous sections as a means of understanding the methods used to test or evaluate each suspect's behavior.

A key conclusion from analyzing all of these weapons is that there is likely no one single weapon that can investigate all variations (“omics”) together simultaneously. Meta-dimensional approaches aim to explore not only genomic features but also proteomic and epigenetic features by integrating the effects of all variations in ingenious ways [94,166]. However, even those approaches cannot be used in isolation, and other methods should be explored to improve our understanding of complex trait architecture. There are obvious strengths and caveats to using each of these approaches which are out of scope of this review to highlight. However, we recommend that, when exploring a new complex disease or trait, multiple suspects are considered along with multiple weapons (or analysis tools) to evaluate the trait of interest thoroughly. It is crucial to evaluate multiple possible analysis strategies for the study of each unique disease or trait.

## 5. Where? Which Tissue(s) Are Important for the Evaluation of Omics Associations?

Every suspect (“variations” as described in previous sections) may alter different tissue types in various biological processes. DNA variations are primarily obtained from blood or serum plasma samples, but it is essential to know which pathway, tissue, or cell type that the risk locus affects. The GTEx database is a powerful resource where the link between gene and the tissue that is affected is investigated [167]. Similarly, for many ocular traits, an ocular tissues database has been utilized [168]. SNPsea is a tool developed at the Broad Institute (Cambridge, MA, USA) to help prioritize the genetic associations to identify tissues and pathways that are affected by the expression of specific genes [169]. Liu et al. utilized SNPsea to identify tissues that are affected by GWAS associations for Systemic Lupus Erythematosus [170] and Hu et al. [171] utilized the same approach to identify tissues affecting the risk loci for autoimmune diseases. Another example where tissue was explored is in an attempt to identify the location of the causes of missing heritability in Type 2 Diabetes, which is one of the most well-studied diseases to identify genetic associations. To identify causal variants and tissues affected by the risk loci, researchers have found that risk associated loci for diabetes and many other metabolic disorders affect adipose tissue as well as observed effects in islets of the pancreas [172,173,174].

In the search for undercover heritability, many suggestions have been made. Genotyping and sequencing technologies rely on the collection of blood samples from study population. Thus, the tissue investigated remains constant in majority of the studies. The common variant common diseases hypothesis (CDCV) suggests that many common variants are the suspects, an additive genetic model is the weapon, and the disease risk manifests in the blood (where the SNPs are measured). Likewise, the rare variant common disease (RVCD) hypothesis suggests that rare variants are the suspects, either burden or distribution of multiple rare variants are the weapons, and blood is the location where the variation is measured. All of these suggestions are tested in one study or the another, but in reality, it seems that we should consider both rare and common variants in multiple possible genetic models for complex diseases. In addition, in elucidating the etiologies of complex traits, the “murder” (or undercover heritability) can take place any location, where location refers to the tissue that is affected by the risk loci. Hence, the secret of this game to identify undercover heritability is to be more open-minded and evaluate each new study with the broad set of data types, tools, and tissues where possible.

## 6. Estimating Heritability (Making a Suggestion in the Game of “Clue”)

The measure of heritability that explains the degree to which a trait is inherited from one generation to another refers to how the heritability values are quantified. The process in which heritability is studied can take place in several different designs as explained below:

- *Family and Twin studies:* In family and twin studies, a set of related individuals and their phenotypic traits are analyzed to identify how heritable the phenotypic trait is in families and in sets of identical twins respectively. Family study estimates are usually lower than twin study estimates. For example, family studies for BMI estimate that BMI is 24–81% heritable whereas twin studies estimate BMI to be 47–90% heritable [34]. The estimates from studies of related individuals take the effects of the environment into consideration and, thus, generally broad sense heritability is estimated by these methods.
- *Population based studies:* Genomic heritability mainly refers to the proportion of trait variance that can be attributable to genetic factors such as common variants and low frequency or rare variations. Many methods and tools exist in the literature to measure heritability among a set of unrelated individuals. These include mixed model approaches (GCTA, REACTA, PLINK, etc.) [175,176], Bayesian approaches (example BGLR) [177], LD based weighted methods in mixed linear model approaches (LDAK) [178], and machine learning approaches (HERRA and MEGHA) [179,180]. All of these methods are focused towards explaining the additive variance component (i.e., narrow sense heritability). Narrow sense heritability estimates from GWAS studies and for either all variants on the genotyping chip assayed or only a subset of statistically significant variants. Locke et al. [181] determined the variance components for BMI based on statistically significant GWAS variants from their study and showed that 97 genome-wide significant loci can explain only 2.7% of the variance, whereas the overall SNP heritability (i.e., heritability from all available genotyped and imputed SNPs) is 75%, as shown by Robinson et al. [182]. Recent studies have also looked at the proportion of phenotypic variance explained by partitioning the genome. Speed et al. [183] showed how the proportion of variance explained for 19 different traits varies across the genome by chromosome and by minor allele frequency ranges. Finucane et al. [184] proposed LD score regression method using summary statistics from GWAS to partition heritability across the genome based on functional annotations.

Methods described above refer to the narrow sense heritability as estimated from GWAS studies which reflects only the additive variance component. With the advent of sequencing technologies, new methods analyzing the effects of rare variants and genetic and gene–environment interactions are also necessary. Heritability estimates for rare variants can be obtained from single variants as well as collapsed regions containing information on the burden of rare variants. To determine unbiased estimates of heritability for gene-based rare variants, Liu et al. [185] proposed a mixed model approach. Next, an estimation of unbiased estimates of interacting variance components remains to be a challenge. Ronnegard et al. [186] estimated genetic marker interactive effects using a ridge regression-based approach. This analysis was conducted in 74 samples from *Arabidopsis thaliana,* but its application on big, large-scale human datasets is still to be explored.

## 7. The Focus of Future Studies, What to Expect?

Addressing a phenotype of interest and analyzing an accurate representation of the phenotype is of great importance. Association analyses are often conducted as retrospective studies, for example by utilizing electronic health record datasets [8], epidemiological datasets [187] and clinical trials datasets [188], among others. Testing of multiple phenotypes simultaneously in PheWAS (Phenome-Wide Association Studies) [189] is also becoming immensely popular, especially with the use of electronic health record (EHR) data and availability of multiple phenotypes. PheWAS provides a significant advantage to test for phenotype relationships (comorbidities and potential pleiotropy) which a single phenotype association study lacks. Occurrences of phenotypes are dependent on many demographic factors such as ancestry, sex, age, etc. Association studies account for these factors in some ways by considering them as confounders and adjusting for their effects. However, it is also crucial to investigate these factors in the context of sub-phenotypes. Verma et al. approached this strategy to address the context of phenotypes in an Aids Clinical Trial Group (ACTG) dataset [190] and identified variants that showed effects on each phenotype of interest in the presence of a drug and in a specific context, where a similar phenotype was not observed in other contexts. This leads to the concept of phenotypic heterogeneity. It is essential to recognize the differences in a phenotype. Association studies aim at understanding a complex phenotype such as type 2 diabetes, cataract, glaucoma, chronic obstructive pulmonary disorder, etc. The analyses of these phenotypes include a single dependent variable which is a phenotype labeled as presence or absence of the disease status for each sample. If we look closer, we observe that these complex phenotypes are a collection of many sub-phenotypes. For example, Li et al. analyzed EHR data to identify patterns among patients and were able to cluster type 2 diabetes patients into three distinct subgroups [191]. Similarly, another study by Verma et al. [192] has shown the importance of incorporating more than one data type from an EHR to identify robust associations. EHRs are highly resourceful as they provide longitudinal information on patients, but utilizing this information is essential and challenging. Longitudinal data tell a patient’s history, and careful assessment of this history can be very fruitful in designing preventive and diagnostic measures. Machine learning methods have been proposed to use longitudinal data from EHR. For example, methods by Zhao et al. [193] and Singh et al. [194] address the issues of temporality from an EHR. Clinical laboratory measures that are part of routine care in health systems are also utilized for association analyses. Historically, one set of values (mean, median, or most recent measurement) values are used. However, clinical measures can provide inferences of a disease diagnosis precisely in the situation where one value for each patient might not tell the complete story. Thus, exploring these longitudinal measures in many different ways such as interquartile ranges (IQR), variance (referring to variability in measures), time-series analysis, and extremely low and high values, etc. can be very beneficial in elucidating the etiology of complex traits.

In this era of rapid molecular technological advancements, we expect to see future studies not looking at the relevance of one omics dataset at a time, but instead considering the combinatorial effects (including additive and interactive) of all possible omics datasets. The field is moving in the direction of integrative modeling approaches (such as meta-dimensional analyses) [166], and, thus, along with detection of risk factors, we should also expect the calculations of variance components attributable by additive, interactive, and environmental effects to be included in the same comprehensive models elucidating undercover heritability. Utilizing a-priori biological information for interpretation of results and identifying interconnected networks affecting disease etiology and what tissues are affected also seems necessary [195]. Many association studies of phenotypes such as lipid traits, cancer, Alzheimer’s disease, and autism to name a few, have been conducted to identify the impact of different genomic variations (suspects) utilizing several analytic methodologies (weapons) to represent their effect in tissues (where) (see Table 2 for example studies in lipid traits).

However, future studies will likely involve the development of methods to integrate all types of omics variations in identifying models affecting disease traits. Methods should also focus on dealing with the sparsity of data because it is highly unlikely to have all of the different data types variations collected on large enough sample sizes. Still, our challenge will be to initiate each new investigation as we would a new round of the board game “*Clue*”. We allow for some variations to classic game of “*Clue*” for one or team of the suspects, weapons, and locations to be the accurate solutions to the crime (disease risk) in question. Another extension to consider in this game is that, in studying the underlying genetic underpinnings of complex traits, we should consider investigating series of crimes and not a single crime. This refers to the concept of phenotypic heterogeneity where many sub-phenotypes (multiple crimes) could be leading to a complex phenotype in investigation. Our ability to explore the different types of omics variation, using different underlying genetic architectures and modeling methods, will be critical to achieving our maximal understanding of the genomics of common, complex diseases and traits.

## Figures and Tables

**Table 1:** A brief list of “weapons” (i.e., models/tools) available to identify genome-phenome associations to uncover heritability. Many of the tools are compiled from Omic Tools resource [112].

Weapon	Suspects	Tool Name	Reference
**Additive Model**	Common variations	PLINK	[113]
	Common variations	PLATO	[114]
	Common variations	QCTool	[115]
	Common variations	GenAbel	[116]
	Common and Rare Variations	BOLT-LMM	[117]
	Common and Rare Variations	FAST-LMM	[118]
	Structural Variations	CNVTools	[119]
	Structural Variations	PennCNV	[120]
	Structural Variations	CKAT	[121]
	Structural Variations	ParseCNV	[122]
	SNPs and Structural Variations	CNVassoc	[123]
	Common and Rare Variations	RVTests	[124]
	Common and Rare Variations	PLINK/SEQ	[125]
	Rare Variations	EPACTS	[126]
	Common variations	MAGMA	[127]
	Rare Variations	EMMAX	[128]
**Gene–Gene Interactions Model**	Common variations	MDR	[129]
	Common variations	AntEpiSeeker	[130]
	Common variations	MultiSURF	[131]
	Common variations	BOOST	[132]
	Common variations	PLATO	[114]
	SNPs and Structural Variations	CNVassoc	[123]
	Common variations	SNPTEST	[133]
	Common variations	TS-GSIS	[134]
	Common variations	SNPAssociation	[135]
	Common variations	PLINK	[113]
	Common Variants and Phenotypes	CAPE	[136]
**Gene-Environment Interactions Model**	Common variations and Environment	PLATO	[114]
**Detecting Heterogeneity**	Genetic variations and Phenotypes	JBASE	[137]
	Gene Expression and Phenotype	SMR	[138]
	Gene Expression and Phenotype	FAST-LMM-EWASher	[139]
	Phenotype	LiCHe	[140]
	Genetic and phenotypic	BUHMBOX	[141]
	Genetic Heterogeneity	ForestPMPlot	[142]
	Genetic variations and Phenotypes	NetDx	[143]
	Genetic Heterogeneity	BioGranat-IG	[144]
**Network based approaches**	SNPs, Phenotypes and Gene Expression	NETAM	[145]
	Common variations	EINVis	[146]
	Gene Expression and Phenotype	NetDecoder	[147]
	Common variations	ViSEN	[148]
	All genetic variations	Cytoscape	[149]
**Pathway analyses**	Common variations	PARIS	[150]
	Genes	SNPSea	[151]
	Genes	GSEA	[152]
	Common variations	VEGAS2Pathway	[153]
	Common variations	MAGENTA	[154]
**Meta-dimensional modelling**	Multi-Omic Datasets	ATHENA	[155]
	Multi-Omic Datasets	NetDX	[143]
	Multi-Omic Datasets	iCluster	[156]
**Gene-based analyses**	All genetic variations	Biofilter	[157]
	Common and Rare Variations	SKAT	[158]
	Rare Variations	BioBin	[159]
	Rare Variations	Variant Association Tools	[68]
	Rare Variations	EPACTS	[126]
**Feature Selection/Prioritization**	All genetic variations	Biofilter	[157]
	Common variations	GLM (LASSO and Elastic-Net)	[160]
	Common variations	RANGER	[161]
	Common variations	Gradient Boosting	[162]
**Causal Variant Determination**	Common variations	TATES	[163]
	Common variation and eQTL	CAVIAR	[164]
	Common variation and eQTL	PrediXcan	[165]

**Table 2:** Examples for use of different methods to exploit genetic architecture of lipid traits.

Analysis Type	References
**Common variants**	Rotroff et al. [196], Ligthart et al. [197]
**Rare Variants**	Liu et al. [185], Surakka et al. [198]
**Gene–Gene Interactions**	Ma et al. [199], De et al. [200], Holzinger et al. [201]
**Gene–Environment Interactions**	Ordovas [202], Shungin et al. [203]
**Gene Expression analysis**	Wen et al. [204]
**Proteomics**	Luczak et al. [205]
**Meta-dimensional analysis**	Holzinger et al. [206]
**Phenotype Heterogeneity**	Morabia et al. [207]
